# An Ultrasensitive Silicon-Based Electrolyte-Gated Transistor for the Detection of Peanut Allergens

**DOI:** 10.3390/bios12010024

**Published:** 2022-01-04

**Authors:** Donghoon Kim, Bo Jin, Sol-A Kim, Wonyeong Choi, Seonghwan Shin, Jiwon Park, Won-Bo Shim, Kihyun Kim, Jeong-Soo Lee

**Affiliations:** 1 Department of Electrical Engineering, Pohang University of Science and Technology (POSTECH), Pohang 37673, Korea; kdong620@postech.ac.kr (D.K.); shengzhi86@postech.ac.kr (B.J.); pathfinder@postech.ac.kr (W.C.); ssh3290a@postech.ac.kr (S.S.); jwpark78@postech.ac.kr (J.P.); 2 Research and Development Department, Innovative General Electronic Sensor Technology Co., Itd. (IGEST), Pohang 37673, Korea; 3 Division of Applied Life Science, Graduate School, Gyeongsang National University, Jinju 52828, Korea; thfdk718@naver.com; 4 Department of Food Science and Technology, Gyeongsang National University, Jinju 52828, Korea; wbshim@gnu.ac.kr; 5 Division of Electronics Engineering, Jeonbuk National University, Jeonju 54896, Korea; 6 Future Semiconductor Convergence Technology Research Center and ICT Convergence Research Center, Jeonbuk National University, Jeonju 54896, Korea

**Keywords:** BioFET, biosensor, electrolyte gate, limit of detection, peanut allergen

## Abstract

The highly sensitive detection of peanut allergens (PAs) using silicon-based electrolyte-gated transistors (Si-EGTs) was demonstrated. The Si-EGT was made using a top-down technique. The fabricated Si-EGT showed excellent intrinsic electrical characteristics, including a low threshold voltage of 0.7 V, low subthreshold swing of <70 mV/dec, and low gate leakage of <10 pA. Surface functionalization and immobilization of antibodies were performed for the selective detection of PAs. The voltage-related sensitivity (*S_V_*) showed a constant behavior from the subthreshold regime to the linear regime. The current-related sensitivity (*S_I_*) was high in the subthreshold regime and then significantly decreased as the drain current increased. The limit of detection (LOD) was calculated to be as low as 25 pg/mL based on *S_I_* characteristics, which is the lowest value reported to date in the literature for various sensor methodologies. The Si-EGT showed selective detection of PA through a non-specific control test. These results confirm that Si-EGT is a high-sensitivity and low-power biosensor for PA detection.

## 1. Introduction

Peanuts are a primary source of IgE-mediated food allergies, along with tree nuts, milk, eggs, soybeans, and fish [1,2]. Peanuts can be a particularly severe threat to sensitive people because they can cause life-threatening anaphylactic shock [3]. Additionally, peanut allergies persist into adulthood in 75% to 80% of cases [4]. Peanut allergens (PAs) constitute approximately 85% of the total peanut protein content, and PA is classified as Arachis hypogaea 1 (Ara h1) to Ara h13 [5,6]. Ara h1 and Ara h3 belong to the cupin superfamily and are the two main allergens that cause allergic reactions in up to 35% to 95% of patients [7]. An allergic person is strictly advised to avoid the consumption of peanut-related products because even a trace of PA can trigger an allergic reaction.

The enzyme-linked immunosorbent assay (ELISA) is the most commonly used method for diagnosing PAs. The ELISA has been used as a versatile platform with high reliability and sensitivity, and it has been demonstrated to detect allergenic proteins in peanuts [8,9,10]. However, this technique is typically very time-consuming and requires expensive laboratory equipment and trained experts. Alternative approaches have been reported, including polymerase chain reaction (PCR), real-time PCR, and PCR-ELISA [11,12,13,14]. These methods detect the peanut ingredients in food using PA DNA amplification. The limit of detection (LOD) is typically a few tenths of a DNA copy per reaction (~0.1 mL) [15]. However, these sensitive label-based PCR techniques have drawbacks similar to those of ELISA. Additionally, the quantitative detection of PAs is challenging based on the lack of a direct correlation between DNA concentrations and allergen concentrations.

To overcome the problems associated with ELISA and PCR methodologies, biologically active field-effect transistors (BioFETs) using nanomaterials or nanostructures have been investigated [16,17]. Such BioFETs rely on the label-free detection of electrical signals modulated by binding target biomolecules to a functionalized sensing surface. Several applications of BioFETs have been demonstrated to detect various biomolecules with higher normalized sensitivity and enhanced LODs [18,19,20,21]. However, the sensing current itself is very small as a result of the use of a nanoscale channel, which requires more complex and expensive signal-processing integrated circuits.

Recently, an electrolyte-gated FET (EGT) was introduced [22,23,24,25,26,27]. The large area of the gate electrode in an EGT can be utilized as a functionalization surface to increase the binding probability of biomolecules, thereby increasing the absolute sensing current.

Here, we demonstrated the ultrasensitive and label-free detection of Ara h1 and Ara h3 using an Si-EGT. Our devices were fabricated using micro- and nano-semiconductor processing techniques. Surface functionalization and immobilization of antibodies were performed for the selective detection of PAs. Voltage- or current-related sensitivities depending on the operation regime were investigated to achieve higher sensitivity and lower power consumption. Improved sensitivities and LOD were achieved in a Si-based EGT operation. Non-specific binding tests were also conducted.

## 2. Materials and Methods

### 2.1. Fabrication of Si-EGTs

**Figure 1:**
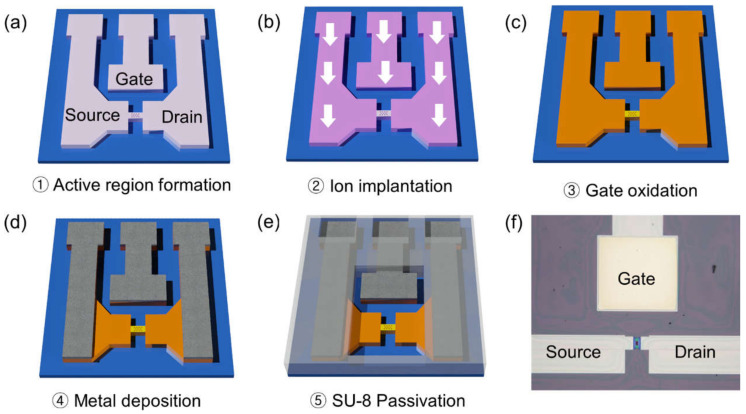
(**a**–**e**) Schematics of fabrication flow and (**f**) an optical image of the top view of the fabricated Si-EGT.

Figure 1 shows the fabrication flow of an Si-EGT using a top-down process. An 8-inch silicon-on-insulator wafer (boron-doped, 10 Ω·cm) with a 100 nm top Si layer and 400 nm buried oxide layer was used as a starting material. The active region (composed of the source, drain, and channel) was defined using deep ultraviolet lithography and an inductively coupled plasma reactive ion etching process. An arsenic dopant with a dose of 2 × 10^15^/cm^2^ was implanted onto the substrate, excluding the channel region. Rapid thermal annealing was then conducted at 1000 °C for 20 s. Next, thermal wet oxidation was applied to grow silicon oxide (5 nm) as a gate insulator. A 50/500 nm Ti/Ag layer was formed using an e-beam evaporator and conventional lift-off process for the contacts, gate electrode, and transmission lines. Finally, an SU-8 passivation layer was formed on the entire surface using photolithography, excluding the gate electrode and channel regions which are supposed to be exposed to the sample solution. The surface areas of the gate electrode and channel were 300 μm × 300 μm and 10 μm × 10 μm, respectively.

### 2.2. Preparation of PAs and Their Antibodies

**Figure 2:**
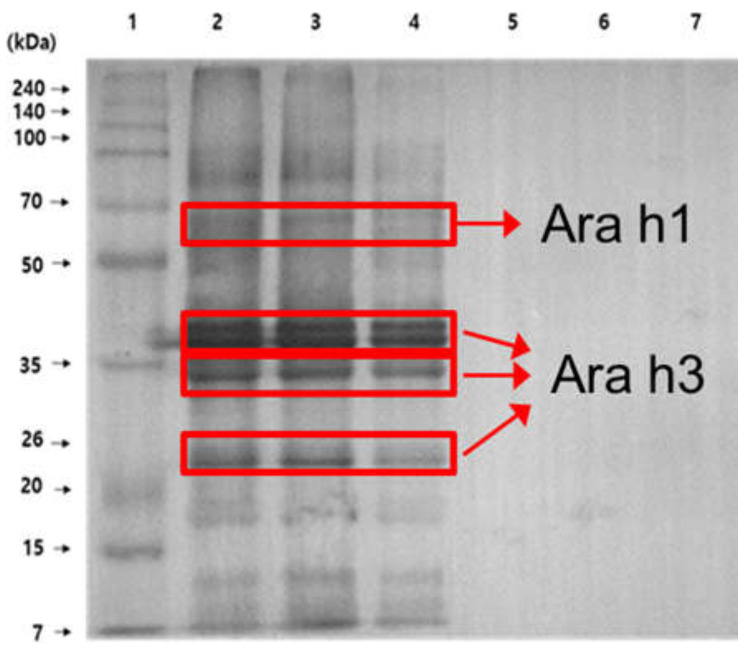
Western blot results. Lane 1: marker; lane 2: roasting peanut; lane 3: boiled peanut; lane 4: peanut butter; lane 5: almond; lane 6: cashew nut; lane 7: red bean.

Monoclonal antibody (Mab) was produced from mice immunized with a thermal-stable soluble protein (TSSP) of peanut. To extract TSSP, including Ara h1 and h3 [28], 1 g of roasted peanut was crushed finely, followed by mixing with 10 mL of carbonate buffer (0.1 M, pH 9.6). The mixture was placed in boiling water for 15 min and then cooled to room temperature. The mixture was then centrifuged at 4 °C for 15 min at 3000 rpm. After filtering the supernatant containing TSSP through Whatman No. 1 filter paper, dialysis in 1 × phosphate-buffered saline (PBS) was performed twice daily for 3 d. The extracted TSSP was used as an immunogen. IgG Mab was developed through cell fusion and cloning [29,30]. Figure 2 shows the Western blot analysis. It confirmed that the antibody was specific to PA without cross-reactivity with other food allergens such as almonds, cashew nuts, and red beans. An antigen-binding fragment (Fab), which is a region on Mab binding to antigens, was generated via papain treatment [31]. After purification using a protein G column, the Fab was used as a receptor to detect PA.

### 2.3. Immobilization of PA Antibodies on Si-EGT

A series of chemical surface treatments were conducted to attach Fab to the sensing surface. First, the devices were rinsed in ethanol and distilled water (DIW) for cleaning, followed by UV/ozone treatment for 1.5 min. Then, 3-aminopropyl-triethoxysilane (APTES) vaporization was performed for 1 min at 50 °C to cover the surface with an amine group (-NH_2_). Next, the device was immersed in a 1 × PBS solution with 2.5% glutaraldehyde for 90 min to form an aldehyde terminal (-CHO) on its surfaces. Finally, the device was exposed to a PA antibody solution (200 μg/mL) for 1 h.

**Figure 3:**
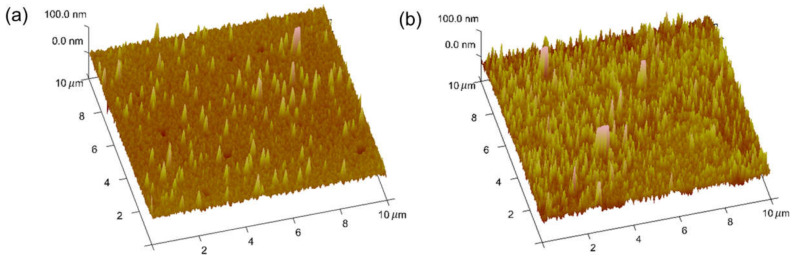
AFM image analysis of the (**a**) bare and (**b**) antibody-immobilized Ag gate electrode.

The surface of the antibody-functionalized Ag electrode was analyzed using atomic force microscopy (AFM, VEECO, New York, NY, USA). As shown in Figure 3a,b, the average roughness and standard deviation were 6 and 9.6 nm for the bare surface, and 10.3 nm and 15.4 nm for the functionalized Ag surface, respectively. The increased surface roughness indicates that the PA antibody was successfully immobilized on the Ag surface.

### 2.4. Electrical Measurement System

The electrical characteristics and PA sensing responses were characterized using a semiconductor parameter analyzer (Keithley 4200SCS, Tektronix, Beavern, OR, USA) at room temperature. The transfer curve (drain current *I_D_* vs. gate voltage *V_G_*) was measured as a function of *V_G_* at *V_D_* = 0.1 V in 20 μL of 0.01 × PBS solution. For quantitative PA analyses, a PA solution using the extracted TSSP in a 1 × PBS solution was prepared with a concentration in the range of 10 pg/mL to 1 mg/mL. After the initial transfer curve was characterized, the device was exposed to 20 μL of PA solution for 20 min, followed by a DIW rinse. Next, 20 μL of 0.01 × PBS solution was dropped onto the device and another measurement was performed to analyze the PA sensing responses.

## 3. Results

### 3.1. Intrinsic Electrical Characteristics

**Figure 4:**
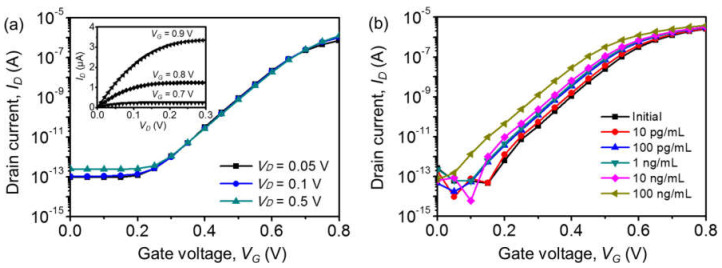
(**a**) Log-scaled *I_D_*-*V_G_* transfer characteristics of the fabricated Si-EGT for various *V_D_*. Inset: *I_D_*-*V_D_* output characteristics. (**b**) Log-scaled *I_D_*-*V_G_* for varying [PA]s of 10 pg/mL to 100 ng/mL.

Figure 4a shows the transfer curves (log (*I_D_*) vs. *V_G_*) and output curves (*I_D_* vs. *V_D_*) of the as-fabricated Si-EGTs prior to surface functionalization. These curves indicate that the device was a typical long-channel n-type FET with a threshold voltage (*V_TH_*) of ~0.7 V. A subthreshold swing (*=* d*V_G_*/dlog(*I_D_*)) of ~70 mV/dec, gate leakage current of <10 pA, and on/off current ratio of ~10^7^ were achieved. The low gate leakage current indirectly confirms the high quality of the gate oxide and successful formation of SU-8 passivation. *I_D_* is exponentially proportional to *V_G_* in the subthreshold regime (*V_G_* < *V_TH_*) and has a linear dependency in the linear regime (*V_G_* > *V_TH_*), as follows [32]:

(1) $$I_{D\_SUB}\;\sim \;\exp[\frac{q(V_{G}\;-\;V_{TH})}{kT}],\;$$

(2) $$I_{D\_LIN}\;\sim \;(V_{G}\;-\;V_{TH}),\;$$

where *q* is the electron charge, *k* is the Boltzmann constant, *T* is the temperature, and *I_D_SUB_* and *I_D_LIN_* are the drain currents in the subthreshold and linear regimes, respectively.

### 3.2. Sensing Responses for PA Detection

Figure 4b shows representative transfer curves for the Si-EGT with various PA concentrations ([PA]s) from the initial PBS (no PA) up to 100 ng/mL. The curve shifts in the negative *V_G_* direction as [PA] increases.

The voltage-related sensitivity (*S_V_*) and current-related sensitivity (*S_I_*) are defined as follows [33,34]:

(3) $$S_{V}=V_{G,Ab}\;–\;V_{G,Ab\_PA},$$

(4) $$S_{I}=\frac{I_{D,Ab}\;-\;I_{D,Ab\_PA}}{I_{D,Ab}},$$

where *V_G,Ab_* and *V_G,Ab_PA_* are the gate voltages after antibody immobilization and antibody–PA reactions at a fixed *I_D_*, respectively, and *I_D,Ab_* and *I_D,Ab_PA_* are the drain currents after antibody immobilization and the antibody–PA reactions at a fixed *I_D_*, respectively.

**Figure 5:**
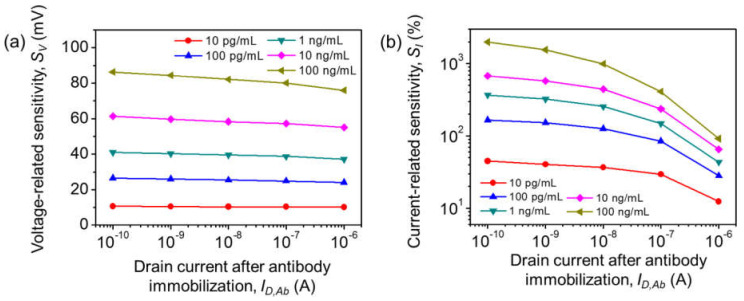
(**a**) Average voltage-related sensitivity *S_V_* and (**b**) average current-related sensitivity *S_I_* vs. *I_D,Ab_* of various [PA]s.

Figure 5a,b show the calculated *S_V_* and *S_I_* as functions of *I_D,Ab_* from the subthreshold to the linear regime. *S_V_* is almost constant, independent of *I_D_*. However, *S_I_* decreases significantly as the device is operated from the subthreshold to linear regimes by increasing *V_G_*. According to Equation (1), *I_D_* increases exponentially with a linear change in *V_G_* in the subthreshold regime. For example, with a PA of 100 ng/mL, the *S_I_* value is as high as 2000% at *I_D,Ab_* = 100 pA (subthreshold regime), which is almost 200 times higher than *S_I_* at *I_D,Ab_* = 1 μA (linear regime). Therefore, the device should be operated in the subthreshold regime to achieve high sensitivity and low power consumption.

### 3.3. Sensitivity, LOD, and Selectivity

**Figure 6:**
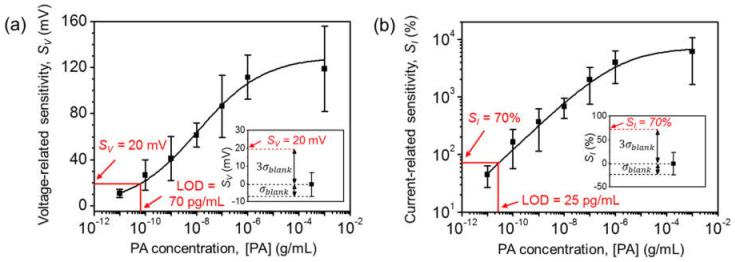
[PA] vs. (**a**) *S_V_* and (**b**) *S_I_* at *I_D,Ab_* = 100 pA. The solid lines which are showed in the figures are logistic fitted lines. Inset: *S_V_*, *S_I_* of blank replicates (1 × PBS solution with no PAs), and *S_V_*, *S_I_* at the LOD using the three-sigma method.

Figure 6a,b show *S_V_* and *S_I_* vs. the logarithm of the [PA] at *I_D,Ab_* = 100 pA. A logistic calibration curve was used to determine the relationship between sensor responses and antigen concentrations [35,36,37]. The curves from the fit were *S_V_* = 130 × [PA]^0.34^/(2.06 × 10^−3^ + [PA]^0.34^) and *S_I_* = 7100 × [PA]^0.42^/(3.33 × 10^−3^ + [PA]^0.42^). The dynamic range was more than four orders of magnitude from 100 pg/mL to 1 μg/mL of [PA].

The LOD was calculated using the three-sigma method [38,39]. The standard deviations from the blank replicate (1 × PBS solution without PA) were as low as 7 mV for *S_V_* and 23% for *S_I_*. The extracted LODs for *S_V_* and *S_I_* were as low as 70 pg/mL and 25 pg/mL, respectively. Table 1 summarizes the LODs of various PA sensors in the literature, including ELISA [9], heat transfer methods [40], surface-enhanced Raman spectroscopy (SERS) [41], and resistors [42,43]. The LOD for our Si-EGT is almost ten times lower than those in previous reports.

**Figure 7:**
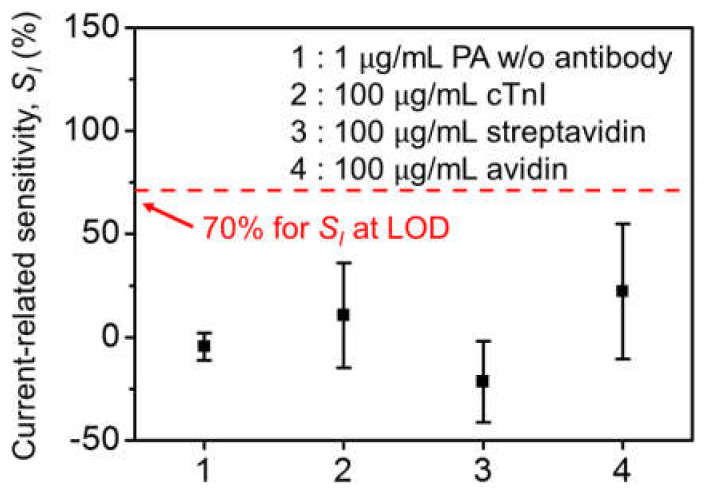
Selectivity test with 1 μg/mL PA without surface functionalization, 100 μg/mL cTnI, streptavidin, and avidin with surface functionalization at *I_D,Ab_* = 100 pA.

Figure 7 shows non-specific control experiments for confirming PA selectivity. The immobilized devices were exposed to cardiac troponin I (cTnI), streptavidin, and avidin. The *S_I_* values from relatively high-concentration biomolecules were far below the LOD level, indicating that the sensitivity stems from specific PA binding. Additionally, *S_I_* was almost zero with no surface functionalization upon exposure to 1 μg/mL of PA, suggesting that the PA antibodies were successfully immobilized.

### 3.4. Detection Principle of Si-EGTs

Binding events on a sensing surface are described using various methods [23,24,25,44]. Among them, the presence of a dipole layer has been typically utilized to explain the detection principle of EGT devices [23,24,25]. The magnitude and direction of the dipole layer are largely dependent on the type of antibody–antigen conjugate and its density on the surface. The dipole potential (*V_DP_*) generated by an antibody–antigen reaction is expressed as [25]:

(5) $$V_{DP}=\frac{N_{Ab\_Ag}\;P_{DP}}{\varepsilon_{DP}},\;$$

where *N_Ab_Ag_* is the areal density of antibody–antigen conjugates, *P_DP_* is the dipole moment, and *ε_DP_* is the dielectric constant of the dipole layer. Because *P_DP_* and *ε_DP_* are intrinsic characteristics of the conjugates and are the same on both gate electrode and the channel, *V_DP_* has a proportional relationship with *N_Ab_Ag_*.

**Figure 8:**
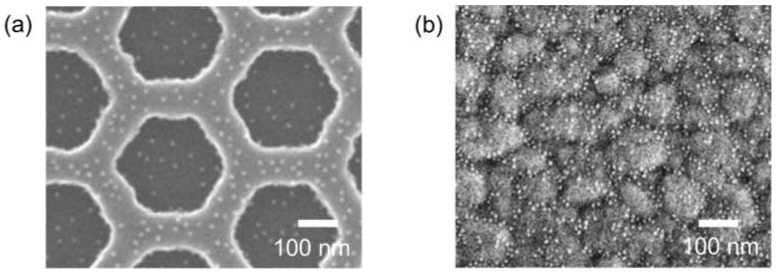
SEM images of (**a**) the channel and (**b**) the gate electrode with the immobilization of AuNPs (diameter: 10 nm).

To compare the induced *V_DP_* on the gate electrode (*V_DP,GT_*) and channel (*V_DP,CH_*), *N_Ab_Ag_* can be estimated using a gold nanoparticle (AuNP)-conjugated antigen. Based on the limited availability of AuNP-conjugated PA, streptavidin and AuNP-conjugated biotin were used alternatively. After the same functionalization treatment, 1 mg/mL of streptavidin solution was exposed for 2 h, followed by a reaction with AuNP-conjugated biotin for 2 h. Figure 8 shows the scanning electron microscopy (SEM) images of the immobilized AuNPs on the channel and the gate electrode. AuNPs were mainly attached along the grain boundary of the gate electrode. The areal density of AuNPs on the gate electrode was 5 to 10 times higher than that on the channel. This result indicates that *V_DP,GT_* is the dominant component affecting the sensing characteristics of Si-EGT.

**Figure 9:**
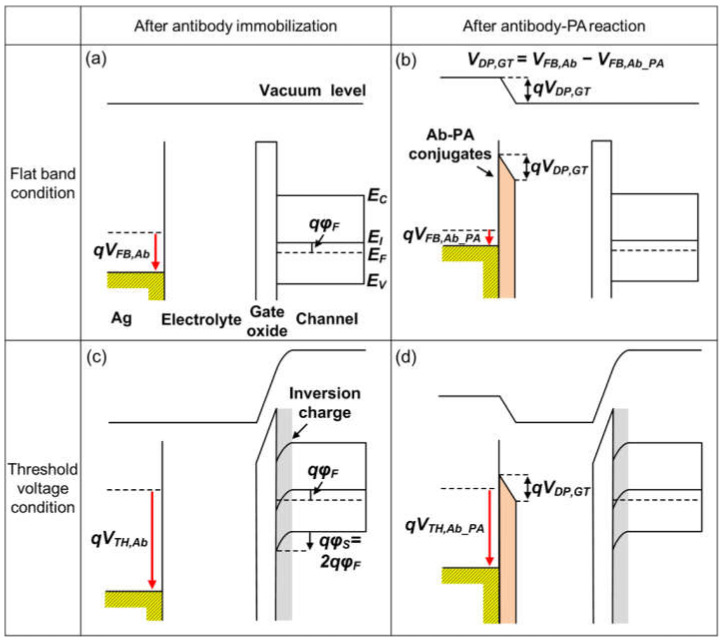
(**a**–**d**) Energy band diagrams of EGTs after antibody immobilization and after antibody–PA reactions under flat band conditions (*V_G_* = *V_FB_*, *φ_S_* = 0) and threshold voltage conditions (*V_G_* = *V_TH_*, *φ_S_* = 2*φ_F_*).

Figure 9 shows an energy band diagram for the Si-EGT after antibody immobilization and antibody–PA reaction. This diagram explains several sensing characteristics with two representative operation points: the flat band condition (*V_G_* = *V_FB_*, *φ_S_* = 0) and threshold voltage condition (*V_G_* = *V_TH_*, *φ_S_* = 2*φ_F_*). The dipole potential on the gate electrode causes a change in the effective work function of the gate metal, which is relevant to the threshold voltage (*V_TH_*) [23,24,25].

When a PA solution is dropped onto the antibody-immobilized Si-EGT, the antibody–PA conjugate forms a dipole layer on the gate surface where the electric field points toward the gate electrode, as shown in Figure 9b,d. The potential drop (*V_DP,GT_*) across the dipole decreases the work function of the gate electrode, followed by *V_FB,Ab_PA_* = *V_FB,Ab_* − *V_DP,GT_*. Therefore, an increase in [PA] requires a less positive *V_G_* to flow the same drain current, or it results in the reduction of *V_TH_*.

## 4. Conclusions

We demonstrated the highly sensitive detection of PAs using Si-based electrolyte-gated FET biosensors. The fabricated devices showed excellent intrinsic characteristics, including a low *V_TH_* of ~0.7 V, low subthreshold swing of ~70 mV/dec, low gate leakage current of <10 pA, and high *I_ON/_I_OFF_* ratio of ~10^7^. The voltage-related and current-related sensitivities depending on the operation regimes were investigated to achieve higher sensitivity and low power consumption. The current-related sensitivity decreases exponentially as *I_D_* increases from the subthreshold regime to the linear regime. The voltage-related sensitivity is almost constant with the gate voltage and increases by approximately 90 mV for [PA] = 100 ng/mL. The calculated LOD is 25 pg/mL. High specificity was also confirmed through non-specific binding tests. These results suggest that Si-EGT is very promising for identifying PAs and screening PAs in food samples.

## Figures and Tables

**Table 1:** LOD comparison between various PA sensors.

Sensor Type	PA Type	Dynamic Range	LOD	Ref.
ELISA	Ara h3	240 pg/mL–125 ng/mL	240 pg/mL	[9]
Heat transfer method	Ara h1	315 ng/mL–3 μg/mL	189 ng/mL	[40]
SERS	Ara h1	90 ng/mL–36 μg/mL	90 ng/mL	[41]
Two electrodes resistor	Ara h1	12.6 ng/mL–2 μg/mL	3.8 ng/mL	[42]
Carbon nanotube resistor	Ara h1	1 ng/mL–100 μg/mL	1 ng/mL	[43]
Si-EGT	Ara h1, h3	100 pg/mL–1 μg/mL	25 pg/mL	This work
